# Tumor Suppressor miR-184 Enhances Chemosensitivity by Directly Inhibiting SLC7A5 in Retinoblastoma

**DOI:** 10.3389/fonc.2019.01163

**Published:** 2019-11-15

**Authors:** Tian-Geng He, Zi-Yun Xiao, Yi-Qiao Xing, Hua-Jing Yang, Hong Qiu, Jian-Bin Chen

**Affiliations:** ^1^Department of Ophthalmology, Tianjin Medical University General Hospital, Tianjin, China; ^2^Department of Funds Disease, Enshi Huiyi Ophthalmology Hospital, Enshi, China; ^3^Eye Center, Renmin Hospital of Wuhan University, Wuhan, China; ^4^Department of Ophthalmology, Tongji Medial College, Tongji Hospital, Huazhong University of Science and Technology, Wuhan, China; ^5^Department of Oncology, Tongji Medial College, Tongji Hospital, Huazhong University of Science and Technology, Wuhan, China

**Keywords:** miR-184, SLC7A5, apoptosis, cell cycle, chemosensitivity, retinoblastoma

## Abstract

The expression patterns and functional roles of miRNAs in retinoblastoma (RB) are poorly understood, especially those involved in chemoresistance. Here, we validated the expression pattern of 20 potential RB-suppressive miRNAs and confirmed that miR-184 is the most significantly decreased miRNA in human RB tissues, as well as chemoresistant cell line. Bioinformatic and molecular analyses revealed that SLC7A5 has three binding sites of miR-184 and significantly increased in RB tissues. miR-184 negatively correlated with SLC7A5 expression in RB tissues and mainly target position 2494-2513 of the SLC7A5 3′UTR to inhibit its expression. Furthermore, enforced expression of miR-184 reversed the oncogenic roles of SLC7A5 on proliferation, migration, and invasion of RB cells. In addition, miR-184 also enhances chemosensitivity of RB cells *via* inducing apoptosis and G2/M cell cycle arrest. Molecular studies revealed that miR-184-decreased phosphorylation status of known DNA damage repair sensors of the ATR/ATM pathways and induced persistent formation of γH2AX foci depend on targeting SLC7A5, leading to persistent DNA damage. Thus, targeting the miR-184/SLC7A5 pathway will provide new opportunities for drug development to reverse chemotherapeutic resistance in RB.

## Introduction

Retinoblastoma (RB) is the most common intraocular malignancy in infants and childhood, with an incidence of 1/15,000–20,000 live births, and mortality from RB is about 70% in developing countries (1, 2). This dismal outcome is mainly attributed to delayed diagnosis with the absence of perceptible pathological change and, more crucially, its inherent propensity of resistance to chemotherapy, which is currently one of the important treatment modalities for RB (3, 4). Therefore, further understanding the molecular mechanism(s) of RB pathogenesis/chemoresistance is urgently needed to improve the clinical therapeutic effects.

Emerging evidences suggests that the dysregulated expression of miRNAs plays important roles in RB to act as either tumor suppressors or oncogenes (e.g., miR-17~92 cluster, miR-34a) (5, 6). Targeting these oncogenic miRNAs, like miR-17~92, and miR-18a inactivation, significantly suppresses RB formation *in vivo*, cosilencing of miR-17/20a and p53 cooperatively decreases the viability of human RB cells (2, 5). As tumor-suppressive miRNAs, miR-183 inhibits the growth, migration, and invasion of RB cells through targeting LRP6 (7); miR-34a-dependent HMGB1 downregulation inhibits autophagy and enhances chemotherapy-induced apoptosis in RB cells (6). It is noteworthy that dysregulation of specific miRNAs leads to chemoresistance in numerous cancers, and correction of these miRNAs using miRNA mimics or antagomiRs can normalize the gene regulatory network and thus sensitize cancer cells to chemotherapy (8–10). miR-34a also increases sensitivity to conventional chemotherapy by directly targeting cancer stemness-related protein, NOTCH1 (11) or GOLPH3 (12), as well as inhibits 53BP1-mediated DNA damage repair (13) in different cancers. Therefore, miRNA-targeted therapy provides an attractive antitumor approach for integrated cancer therapy.

However, miR-34a is increased 3–100 folds in RB patient samples, contrary to RB cell lines and other cancers (5, 14). miR-17~92 cluster is downregulated in chemoresistant cancer stem cells (CSCs) of pancreatic cancer, and overexpression of miR-17~92 cluster enhances chemosensitivity of CSCs by targeting multiple Nodal/Activin/TGF-β1 signaling cascade members (15). In addition, as an important tumor-suppressive miRNA, let-7b also enhances chemosensitivity of cancer cells (16, 17) and is significantly decreased in human RB samples (5, 18). Although some progress has been achieved in the past decades, the expression patterns and functional roles of miRNAs in RB still needed to be further elucidated, particularly the role of miRNAs in RB chemoresistance (18).

Here, we validated the expression pattern of 20 potential RB-suppressive miRNAs (5, 18) and confirmed that miR-184 significantly decreased in human RB tissues, as well as chemoresistant cell lines. Molecular studies further determined that enforced miR-184 plays tumor-suppressive roles in RB cells and enhances chemosensitivity *via* enhancing G2/M phase arrest and cellular apoptosis mediated through directly targeting SLC7A5 and its downstream ATR/ATM pathway.

## Materials and Methods

### Human Tissue Samples and Cell Culture

Fifteen paraffin-embedded human RB tissues and three normal retina tissues were collected from Tianjin Medical University General Hospital, Ensure Huiyi Ophthalmology Hospital and Tongji Hospital (Wuhan, China), under approval of the institutional review board, and written informed consent was obtained from all subjects. The human RB cell lines WERI-RB1, Y79, and Y79/EDR [etoposide (ETO)-resistant] were cultured in RPMI 1640 medium (HyClone, USA) supplemented with 10% heat-inactivated fetal bovine serum (FBS, Life Technologies), 100 U/ml penicillin, and 100 μg/ml streptomycin (Beyotime, Shanghai, China) in a humidified atmosphere at 37°C with 5% CO_2_. The cells in the exponential phase of growth were used in the experiments.

### Y79/EDR Cell Line

ETO-resistant Y79 cell line Y79/EDR was established by culturing Y79 cells with increasing concentrations of ETO (from 1 to 500 nM) for 6 months and then maintained in the absence of drug for 2 weeks. The IC_50_ was determined by measuring viability using CCK-8 assay (19).

### EdU Assay

Cell proliferation assay was performed using the BeyoClick™ EdU Cell Proliferation Kit with Alexa Fluor 647 (Beyotime). Briefly, the cells were seeded in 96-well plates at a density of 5 × 10^3^ cells/well for 48 h after transfection and then treated with indicated drugs. Then, the cells were incubated with 10 μM EdU for 2 h at 37°C. After being fixed with 4% paraformaldehyde for 30 min, the cells were treated with 0.1% Triton X-100 for 10 min and rinsed with PBS three times. Thereafter, the cells were exposed to 100 μl of click reaction cocktail for 30 min and then incubated with 5 μg/ml of Hoechst 33342 to stain the cell nuclei for 30 min. Images were captured using Olympus IX73 microscope. The percentage of EdU-positive cells was defined as the proliferation rate. All the experiments were performed in triplicate.

### Cell Transfection

SLC7A5 siRNAs (siR1 and siR2), human miR-184 mimic, inhibitor, and their corresponding negative controls (NC) were synthesized from RiboBio (Guangzhou, China). SLC7A5 mRNA CDS region (GenBank accession no. NM_003486.6) was amplified from normal human retina tissue and inserted into the pcDNA3.1 vector (Invitrogen, Shanghai, China). Transfection was performed with Lipofectamine 3000 (Invitrogen) following the manufacturer's protocol. Forty-eight hours after transfection, the selective silencing and overexpression performance were identified by qRT-PCR or Western blotting.

### Luciferase Assay

SLC7A5 3′-UTR including three putative binding sites of miR-184 was amplified by PCR from cDNA of normal retina. DNA sequences (about 200 bp) containing each miR-184 binding site and its paired site-specific mutation (wt1/mut1, wt2/mut2, wt3/mut3) on the 3′UTR of SLC7A5 were synthesized by TsingKe Biotech (Wuhan, China) and subsequently cloned into the dual luciferase vector psiCHECK2 (Promega, Beijing, China). Y79 cells plated in 24-well plates were cotransfected with 500 ng of the reporter recombinant constructs and 30 nM of miR-184 mimic or miR-NC. After 48 h, cells were lysed and analyzed for Renilla and Firefly luciferase activity using the Dual-Glo luciferase assay system (Promega). All experiments were performed in triplicate. Activity of Renilla luciferase was normalized to Firefly luciferase.

### qRT-PCR

Total RNA of cells was extracted using Trizol (Invitrogen), and RNA of paraffin-embedded tissues was extracted using FFPE RNA extraction kit (AmoyDx, Xiamen, China) according to the manufacturer's instruction. For miRNA expression analysis, 10 ng of total RNA from each sample was transcribed into cDNA using TaqMan® MicroRNA Reverse Transcription kit (Applied Biosystems, Shanghai, China). qRT-PCR was performed using TaqMan® 2× Universal PCR Master Mix no UNG (Applied Biosystems), and primers for miRNAs and U6 were synthesized from RiboBio. To analyze mRNA expression, both RNA reverse transcription and qRT-PCR amplification were performed using the ABI 7500 Real-Time PCR System with ReverTra Ace-α First-strand cDNA Synthesis kit and SYBR Green real-time PCR Master Mix kit (Toyobo, Tokyo, Japan), respectively. The primers used for SLC7A5, BIRC3, FLIP, IKB-α, XIAP, BCL-XL, p27, p21, Bim, Bid, Bak, and GAPDH were synthesized from TsingKe Biotech. Each experiment was performed in triplicate. The date was analyzed by the 2^−ΔΔCt^ method.

### Immunofluorescence Detection of γ-H2AX Foci

Forty-eight hours after transfection, Y79 cells were plated in 6-well plate and then treated with ETO (1 μM) for 24 h. Cells were then fixed in 4% paraformaldehyde for 30 min, permeabilized in 0.1% Triton X-100 for 15 min, and then blocked overnight with 1% bovine serum albumin (BSA) at 40°C. Cells were probed with mouse mAb against phosphorylated H2AX Ser139 (γH2AX, CST, USA), washed with Phosphate Buffered Saline (PBS), and probed with Cy3-conjugated goat anti-mouse secondary Ab (Jackson ImmunoResearch, PA, USA). The cells were mounted with 10 μl of ProLong (R) Gold antifade reagent with DAPI (Invitrogen). γH2AX foci were visualized under Olympus IX73 microscope. γH2AX foci were counted in least 100 randomly selected cells per sample.

### Western Blot

Cells were seeded in 6-well plates and treated with indicated conditions. At the indicated time, they were lysed with RIPA buffer (Beyotime). Next, equal amounts of proteins were loaded to sodium dodecyl sulfate-polyacrylamide gel electrophoresis (SDS-PAGE) and then transferred to polyvinylidene fluoride (PVDF) membrane (Millipore, USA). The membranes were blocked with 5% non-fat dry milk in Tris-Buffered Saline Tween-20 (TBST) buffer for 1 h and then probed with primary antibodies at 4°C overnight, followed by the horseradish peroxidase (HRP)-conjugated secondary antibodies. Protein expression was visualized using the enhanced chemiluminescence system (Millipore). Anti-SLC7A5 and anti-TNPO2 antibodies were obtained from Abcam (Shanghai, China). All other antibodies were purchased from CST (Shanghai, China). HRP-conjugated secondary antibodies were purchased from Affinity Biosciences Inc. (Beverly, USA).

### Flow Cytometry

For cell cycle analysis, cells were trypsinized, washed in PBS, fixed in 70% ice-cold ethanol, and stored at −20°C overnight. Samples were then resuspended in PBS and stained with 50 μg/ml propidium iodide (PI) solution containing 0.2% Triton X-100 and 100 μg/ml DNase-free RNase A for analysis. For apoptosis analysis, cells were harvested and stained using Annexin V/PI apoptosis detection Kit (BD Biosciences) according to the manufacturer's instructions. Cells were analyzed by using FACSCalibur (Becton Dickinson). Data analysis was performed using FlowJo version 7.6.1 software (TreeStar).

### Statistical Analysis

Results were expressed as mean ± SD of at least three independent experiments. Unpaired *t*-test or one-way ANOVA followed by Neuman-Keuls *post hoc* test was performed for data analysis, and *p* < *0.05* was considered statistically significant. All statistical analyses were performed using GraphPad Prism version 6.00 (GraphPad Software, Inc.).

## Results

### miR-184 Is a Chemosensitive miRNA

**Figure 1:**
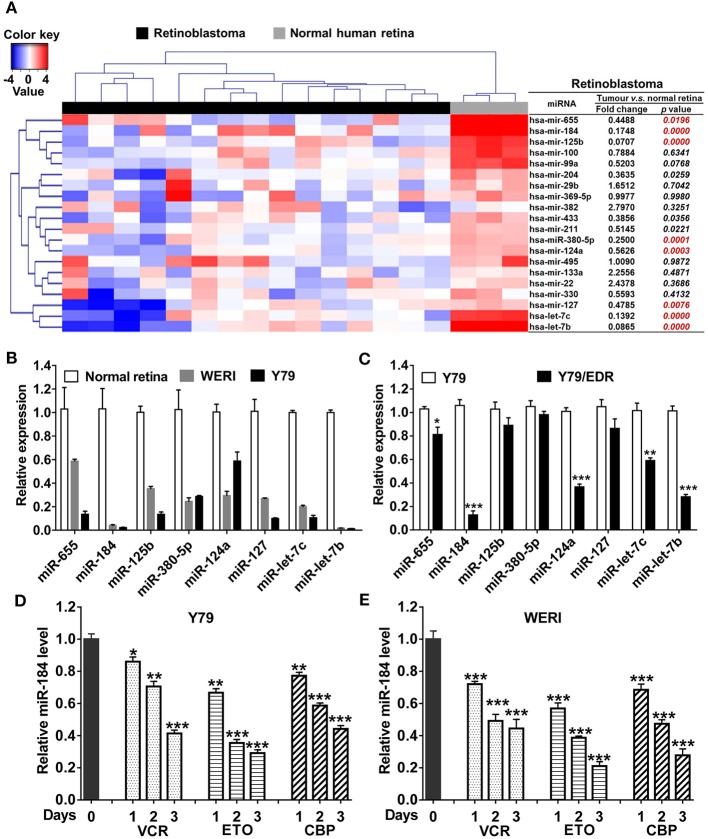
miR-184 is a chemosensitive miRNA in retinoblastoma (RB). **(A)** Expression heatmap of top 20 decreased miRNAs identified by miRNA array in previous studies, which were selected and detected by qRT-PCR in 15 human RB tissues. **(B)** Expression of miRNAs in normal retina (three samples), WERI, and Y79 cells was detected by qRT-PCR. **(C)** Expression of miRNAs in Y79 and Y79/EDR was detected by qRT-PCR. qRT-PCR of miR-184 expression in Y79 cells **(D)** and WERI cells **(E)** treated with VCR (2.5 nM), ETO (0.25 μM), or CBP (1 μM). Data were presented as mean ± SD of three independent experiments. ^*^P < 0.05, ^**^P < 0.01, ^***^P < 0.0001 vs. control group.

To verify the miRNA expression patterns in RB, top 20 decreased miRNAs identified by miRNA array in previous studies (5, 18) were selected and detected in 15 human RB tissues. Compared with normal retina tissues, eight miRNAs were significantly decreased in RB tissues (Figure 1A) and cell lines (Figure 1B), including miR-655, miR-184, miR-125b, miR-380-5p, miR-124a, miR-127, let-7c, and let-7b. Then, these miRNA expression levels in Y79/EDR cells were further detected to investigate their contribution in chemoresistance. miR-184 in Y79/EDR cells was expressed about 8-fold lower than parent Y79 cells and was the most significantly decreased miRNA (Figure 1C). Furthermore, inhibition of miR-184 expression was rapid, starting at day 1 on exposure of Y79 (Figure 1D) and WERI cells (Figure 1E) in culture to vincristine (VCR), ETO, and carboplatin (CBP), which are agents commonly used systemically in RB treatment. These results indicate that miR-184 may function as a tumor-suppressive miRNA and regulate chemosensitivity of RB cells.

### miR-184 Plays Tumor-Suppressive Roles and Enhances Chemosensitivity in RB Cells

**Figure 2:**
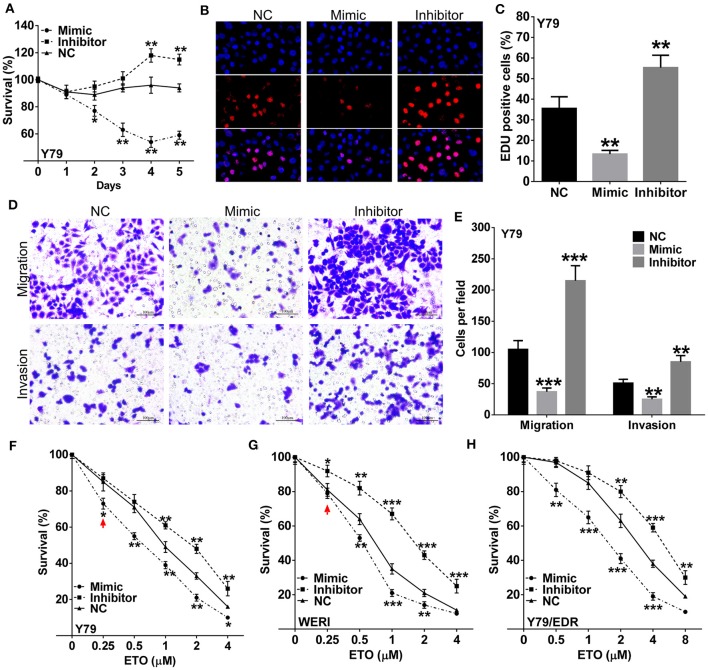
miR-184 is a tumor-suppressive miRNA and enhances chemosensitivity in retinoblastoma (RB) cells. **(A)** Y79 cells were transfected with miR-184 mimic, inhibitor, or negative control (NC). Cell viability was detected by methyl thiazolyl tetrazolium (MTT) assay. **(B)** Y79 cells were transfected with miR-184 mimic, inhibitor, or negative control (NC). Cell viability was detected by 5-Ethynyl-'-deoxyuridine (EdU) assay after 48 h. **(C)** Statistical analysis of the EdU-positive cell ratio in Y79 cells with different transfection. **(D)** Transwell assay of the migration and invasion ability inY79 cells with different transfection. Scale bar: 100 μm. **(E)** Statistical analysis of the cell numbers through the chamber in Y79 cells with different transfection. Y79 cells **(F)**, WERI cells **(G)**, and Y79/EDR cells with different transfection were treated with different concentrations of ETO for 48 h. **(H)** Cell viability was detected by MTT assay. Data were presented as mean ± SD of three independent experiments. ^*^P < 0.05, ^**^P < 0.01, ^***^P < 0.0001 vs. negative control group.

To further characterize the effects of miR-184 in RB, we used miR-184 mimic or inhibitor to modulate cellular levels of miR-184 (Figure S1A). Cell proliferation was significantly decreased by overexpressing miR-184 and enhanced by interfering miR-184 in Y79 (Figures 2A–C) and WERI cells (Figures S1B,C). Cell migration and invasion assays showed that miR-184 mimic inhibited migration and invasion, while miR-184 inhibitor promoted these activities of tumor cells (Figures 2D,E; Figures S1D,E). To investigate whether miR-184 was involved in chemoresistance, Y79 and WERI cells were treated with different concentrations of ETO after miR-184 mimic or inhibitor transfection. By CCK-8 assay, ETO was more cytotoxic in cells transfected with miR-184 mimic and more resistant in miR-184 inhibitor-treated cells (Figures 2F,G). Consistent with this observation, miR-184 also enhanced chemosensitivity of Y79/EDR cells to ETO treatment (Figure 2H). Together, this body of data supporting an increased oncogenicity of miR-184-downregulated RB cells, and miR-184 inhibition enhanced chemoresistance of RB cells.

### miR-184 Enhances Chemosensitivity of RB Cells by Inducing Cell Apoptosis and G2/M Phase Arrest

**Figure 3:**
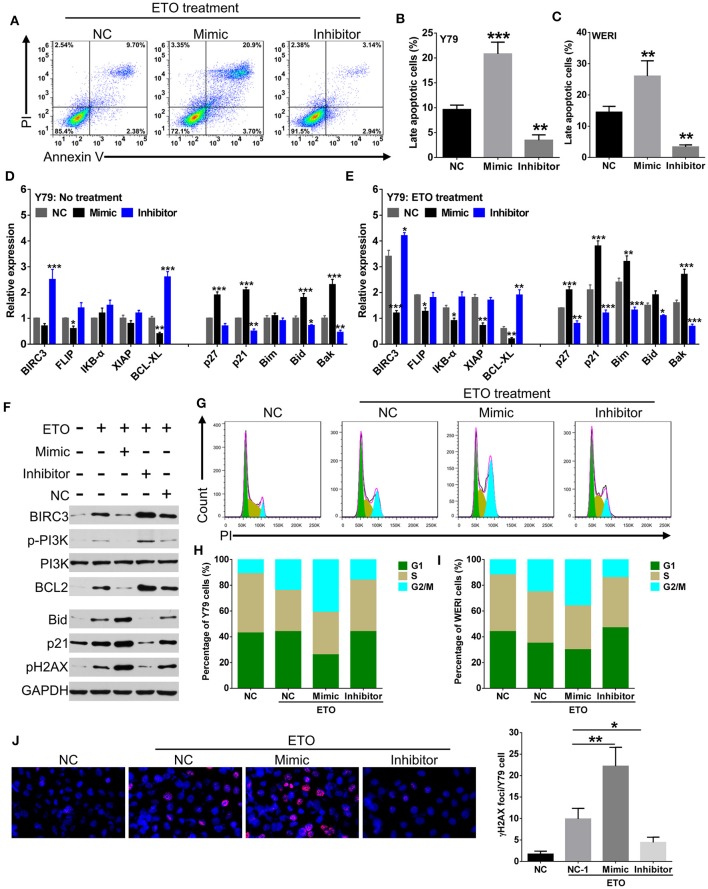
miR-184 enhances apoptosis and G2/M phase arrest of retinoblastoma (RB) cells in response to etoposide (ETO) treatment. **(A)** Y79 cells with different transfection were treated with ETO (0.25 μM) for 48 h. Cellular apoptosis was detected by flow cytometry. Statistical analysis of the Annexin V^+^PI^+^-positive cell ratio in Y79 cells **(B)** and WERI cells **(C)**. Same as in **(A)**, apoptosis-related mRNA expressions in Y79 cells with different transfection alone **(D)** or additionally treated with ETO **(E)** were detected by qRT-PCR. **(F)** Western blot analysis of the expression of apoptosis-related proteins in Y79 cells with indicated treatment same as in **(A)**. **(G)** Cell cycle distribution of Y79 cells with treatment same as in **(A)** was detected by flow cytometry. Statistical analysis of cell cycle phase ratio in Y79 cells **(H)** and WERI cells **(I)**. **(J)** γH2AX foci in Y79 cells with indicated treatment same as in **(A)** was detected by immunofluorescence. Statistical analysis of γH2AX foci in Y79 cells, which were counted in at least 100 randomly selected cells per sample. Data were presented as mean ± SD of three independent experiments. ^*^P < 0.05, ^**^P < 0.01, ^***^P < 0.0001 vs. negative control group.

Induction of apoptosis and cell cycle arrest have been identified as major mechanisms that contribute to enhanced chemosensitivity (20). Susceptibility to the chemodrugs was reflected by an increased level of late-stage apoptosis in miR-184 mimic-transfected cells and a decrease in inhibitor-transfected cells with the presence of 0.25 μM ETO treatment (Figures 3A–C). qRT-PCR analysis revealed that miR-184 inhibited antiapoptotic and enhanced proapoptotic mRNA expression in Y79 cells (Figure 3D) and WERI cells (Figure S2A) and further intensified by ETO treatment (Figure 3E; Figure S2B). Western blotting analysis of Y79 cells in response to ETO treatment also showed that antiapoptotic genes, BIRC3, p-PI3K, and BCL2 levels were decreased, whereas proapoptotic genes, p21, and BID levels were increased by miR-184 (Figure 3F). In addition, exposure to ETO dramatically activated G2 checkpoint and resulted in G2/M phase arrest in Y79 cells (Figures 3G,H) and WERI cells (Figure 3I). miR-184 inhibitor treatment significantly attenuated G2/M phase arrest induced by ETO, whereas miR-184 mimic augmented G2/M phase accumulation induced by ETO (Figures 3G–I). Furthermore, miR-184 mimic increased the formation of γH2AX foci, which represent unrepaired DNA double-strand breaks (DSBs) (21), whereas this process was significantly decreased by miR-184 inhibitor in ETO-treated Y79 cells (Figure 3J). Together, these results indicate that induction of apoptotic cell death and G2/M phase arrest are involved in the mechanisms of chemosensitization by miR-184.

### miR-184 Directly Targets SLC7A5 in RB

**Figure 4:**
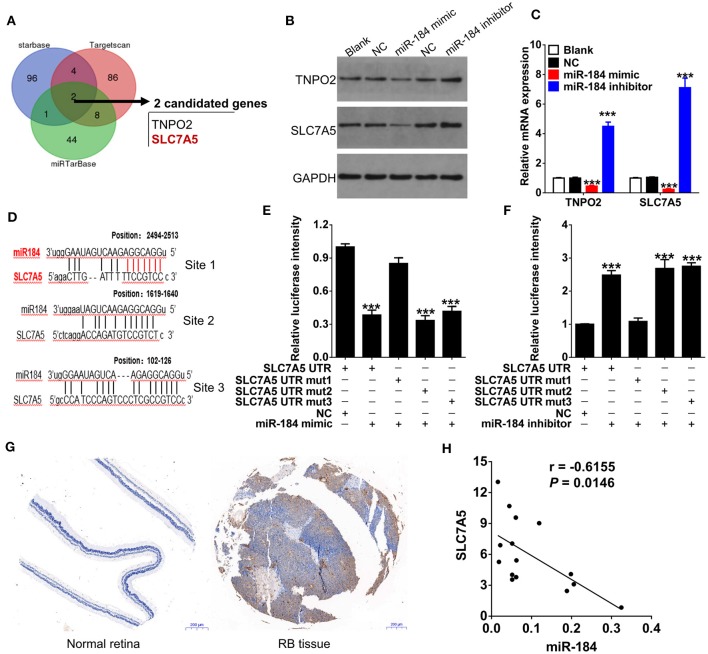
SLC7A5 is a direct target of miR-184. **(A)** Bioinformatics algorithms (TargetScan, Starbase, and miRTarBase) predicted the target of miR-184. Venn diagram found that TNPO2 and SLC7A5 are two shared targets of miR-184. The expressions of SLC7A5 and TNPO2 in Y79 cells with different transfection were detected by Western blot **(B)** and qRT-PCR **(C)**. **(D)** The three putative miR-184 binding sites in 3′UTR of SLC7A5. **(E)** The wild-type (WT) or mutant (Mut) reporter constructs was cotransfected with negative control or miR-184 mimic into Y79 cells, and the dual luciferase activity was determined at 48 h after transfection. **(F)** The WT or Mut reporter constructs were cotransfected with negative control or miR-184 inhibitor into Y79 cells, and the dual luciferase activity was determined at 48 h after transfection. **(G)** SLC7A5 expression in normal retina and Rb tissues was detected by immunohistochemistry. **(H)** Spearman correlation analysis showed that SLC7A5 mRNA level is negatively associated with miR-184 in 15 RB tissues. Data were presented as mean ± SD of three independent experiments. ^***^P < 0.0001 vs. negative control group.

We had thus far observed that miR-184 induced apoptosis and G2/M phase arrest of RB cells in response to chemotherapy, to further unveil the detailed molecular mechanisms, we therefore used multiple bioinformatics algorithms (TargetScan, Starbase, and miRTarBase) and found that TNPO2 and SLC7A5 are two predicted targets of miR-184 (Figure 4A). Thus, we determined the effect of miR-184 on the expression of TNPO2 and SLC7A5 in Y79 cells. miR-184 significantly decreased protein (Figure 4B) and mRNA (Figure 4C) levels of these two genes, and more obvious for SLC7A5, which may be owing to that the 3′UTR of SLC7A5 have three binding sites of miR-184 (Figure 4D). To clarify which binding site or all are responsible for miR-184-mediated inhibition of SLC7A5 expression, the 3′UTR reporter activity of SLC7A5 was further assessed by luciferase assays. Results revealed that miR-184 mimic mainly depended on targeting the first binding site (site 1, position: 2494-2513) of the SLC7A5 3′UTR and leads to a significant decrease in luciferase activity compared with the miR-NC (Figure 4E). Furthermore, this result was further confirmed by miR-184 inhibitor treatment, as when the first binding site was mutated, the effect of miR-184 inhibitor almost disappeared (Figure 4F). Meanwhile, SLC7A5 was strongly upregulated in RB tissues (Figure 4G), and mRNA level of SLC7A5 was inversely correlated with miR-184 (Figure 4H). Thus, these findings suggest that SLC7A5 is the target of miR-184.

### miR-184 Depends on Inhibition of SLC7A5 to Perform Tumor-Suppressive Functions

**Figure 5:**
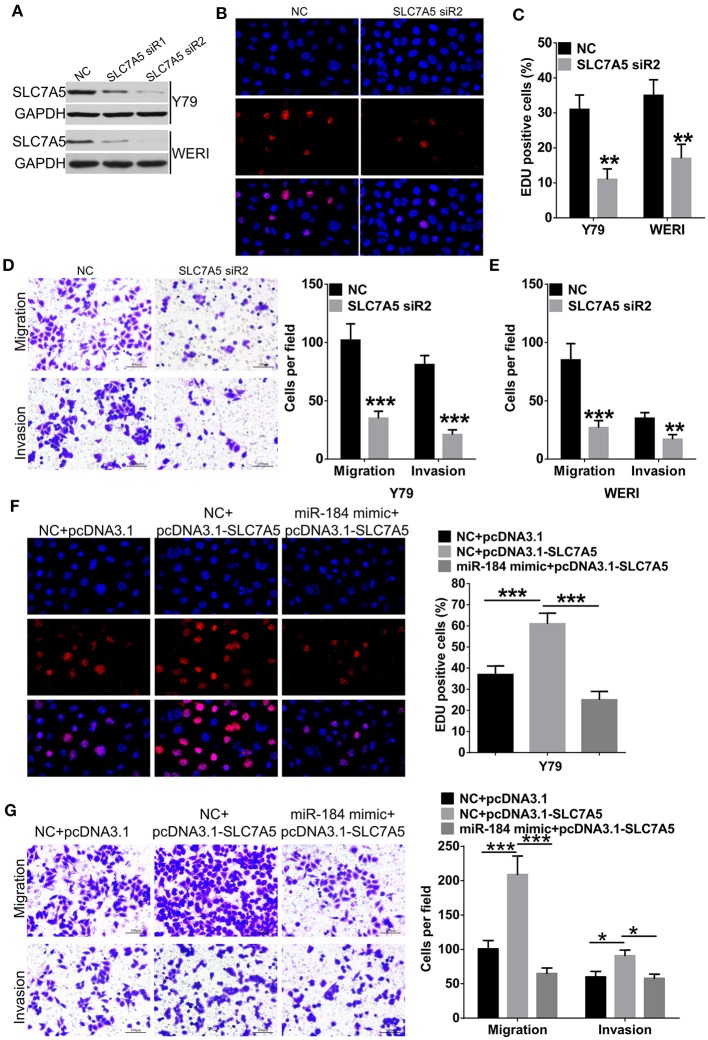
miR-184 inhibits proliferation, migration, and invasion of retinoblastoma (RB) cells *via* targeting SLC7A5. **(A)** Western blot analysis of SLC7A5 expression in Y79 cells and WERI cells transfected with SLC7A5 siRNA. **(B)** EdU analysis of the cell proliferation ability in Y79 cells transfected with the SLC7A5 siRNA. **(C)** Statistical analysis of the EdU-positive cell ratio in Y79 cells and WERI cells transfected with the SLC7A5 siRNA. **(D)** Transwell assay of the migration and invasion ability inY79 cells transfected with the SLC7A5 siRNA. Scale bar: 100 μm. **(E)** Statistical analysis of the cell numbers through the chamber in WERI cells transfected with the SLC7A5 siRNA. **(F)** EdU analysis of the cell proliferation ability in Y79 cells transfected with miR-184 mimic alone or together with SLC7A5 expression vector (pcDNA3.1-SLC7A5). **(G)** Transwell assay of the migration and invasion ability in Y79 cells transfected with miR-184 mimic alone or together with SLC7A5 expression vector (pcDNA3.1-SLC7A5). Data were presented as mean ± SD of three independent experiments. ^*^P < 0.05, ^**^P < 0.01, ^***^P < 0.0001 vs. negative control group.

**Figure 6:**
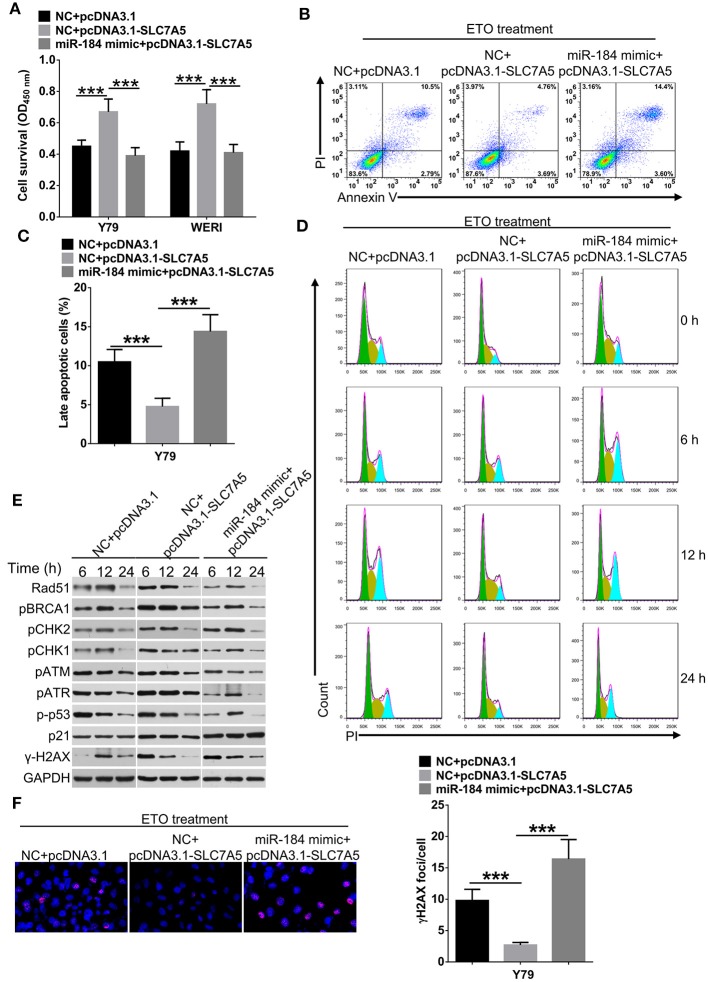
miR-184 enhances apoptosis and G2/M phase arrest of retinoblastoma (RB) cells in response to etoposide (ETO) treatment *via* inhibiting SLC7A5. **(A)** Y79 cells transfected with miR-184 mimic alone or together with SLC7A5 expression vector (pcDNA3.1-SLC7A5) were treated with ETO (0.25 μM) for 48 h. Cell survival was detected by MTT assay. **(B)** Y79 cells transfected with miR-184 mimic alone or together with SLC7A5 expression vector (pcDNA3.1-SLC7A5) were treated with ETO (0.25 μM) for 48 h. Cellular apoptosis was detected by flow cytometry. **(C)** Statistical analysis of the Annexin V^+^PI^+^-positive cell ratio in Y79 cells. **(D)** Forty-eight hours after transfection with miR-184 mimic alone or together with SLC7A5 expression vector (pcDNA3.1-SLC7A5), Y79 cells were treated with ETO (0.25 μM), and cell cycle distribution was detected by flow cytometry at each time point. **(E)** Forty-eight hours after transfection with miR-184 mimic alone or together with SLC7A5 expression vector (pcDNA3.1-SLC7A5), Y79 cells were treated with ETO (0.25 μM), and expressions of DNA damage sensor proteins in the ATM/ATR pathways were detected by Western blot at each time point. **(F)** Forty-eight hours after transfection with miR-184 mimic alone or together with SLC7A5 expression vector (pcDNA3.1-SLC7A5), Y79 cells were treated with ETO (0.25 μM) for 24 h, and γH2AX foci were detected by immunofluorescence. Statistical analysis of γH2AX foci in Y79 cells, which were counted in at least 100 randomly selected cells per sample. Data were presented as mean ± SD of three independent experiments. ^***^P < 0.0001 vs. negative control group.

Overexpression of SLC7A5 has been observed in a wide range of tumor cells (22). In RB cells, knockdown of SLC7A5 (Figure 5A) significantly inhibited proliferation (Figures 5B,C), migration and invasion (Figures 5D,E). Meanwhile, overexpression of SLC7A5 (Figure S3A) promoted these activities (Figures 5F,G; Figures S3B,C), and which could be blocked by simultaneously cotransfecting with miR-184 mimic. Furthermore, miR-184 mimic also blocked the promotive effects of exogenous SLC7A5 expression on survival (Figure 6A) and induced late-stage apoptosis (Figures 6B,C; Figure S3D) of RB cells in the presence of ETO.

To avoid DNA damage induced by chemodrugs, tumor cells temporarily stop cell cycle progression, allowing the repair of the lesions (23). Therefore, we analyzed whether miR-184 depended on SLC7A5 to regulate cell cycle dynamics in Y79 cells upon ETO treatment. As shown in Figure 6D and Figure S3E, SLC7A5-overexpressing cells showed a significant accelerated and shortened response of cell cycle to ETO, with the highest G2/M arrest after 6 h and returned to normal after 12 h, whereas control Y79 cells had the highest fraction of G2/M phase arrest at the 12-h time point and returned back to normal G1/S/G2 distribution after 24 h. Importantly, cotransfection of miR-184 mimic significantly prolonged cell cycle progression of SLC7A5-overexpressing cells in response to ETO treatment. The ATR/ATM pathway is crucial for the survival of tumor cells in responding to DNA replication stress and DNA damage (24). A time-course analysis revealed that SLC7A5 significantly increased the phosphorylation status of known DNA damage repair sensors in ATR/ATM pathway since 6 h post ETO treatment (Figure 6E), whereas cotransfection of miR-184 mimic significantly suppressed the activation of ATR/ATM pathway induced by SLC7A5 and induced persistent γ-H2AX expression after ETO treatment in Y79 cells (Figures 6E,F). Taken together, these findings suggest that miR-184 performs tumor-suppressive roles and enhances chemosensitivity *via* directly targeting SLC7A5.

## Discussion

Although miR-34 family (miR-34a, b, c) plays tumor-suppressive and chemosensitive roles, its expression level in human or mice RB tissues is inconsistently by profiling miRNA expressions (5, 6, 25). In addition, the function of miR-17~92 cluster in RB also exhibits inconsistency with adult cancers (5, 15). Thus, we speculated that the expression pattern and function of miRNAs in childhood-specific RB may differ from adult cancers and need to be further clarified. Here, we measured the expression pattern of top 20 potential RB-suppressive miRNAs identified by microarray (5, 18) and confirmed that miR-184 is the most significantly decreased miRNA in human RB tissues, as well as chemoresistant cell line. Furthermore, contrary to a potential oncogenic miRNA in squamous cell carcinoma of the tongue (26), enforced expression of miR-184 significantly inhibited proliferation, migration, and invasion while promoting apoptosis and inducing G2/M phase arrest in RB cells.

Previously, miR-184 has been reported to inhibit malignant glioma progression *via* directly targeting SND1 (27) and serves as a tumor suppressor in breast cancer by directly targeting multiple proteins involved in PI3K/AKT/mTOR pathway (28). Unfortunately, overexpression of these proteins (like AKT2, PRAS40, or GSK3A) does not rescue miR-184 repression of protein synthesis, highlighted the existence of other target genes of miR-184 in PI3K/AKT/mTOR pathway (28). In this study, bioinformatic and molecular analysis revealed that miR-184 directly targeted 3′UTR of SLC7A5 to inhibit its expression. As a critical amino acid transporter, SLC7A5 was mainly involved in regulating proliferation, apoptosis, and chemoresistance of cancer cells through activating the downstream AKT/mTOR pathway (29, 30). Cellular and molecular studies further demonstrate that SLC7A5 has three binding sites of miR-184, and targeting SLC7A5 with miR-184 significantly blocks its downstream cellular effects and enhances the anti-RB effects of chemotherapeutic drugs *in vitro*. Thus, SLC7A5 is an important additional gene for miR-184 to inhibit the activation of the PI3K/AKT/mTOR pathway.

Mounting evidence has confirmed that SLC7A5 can be upregulated by c-myc, ATF4, and HIF-2α in an mTORC1-dependent mechanism, leading to increased uptake of essential amino acids (e.g., L-Leu), and thus further activating mTORC1 pathway to form a vicious positive feedback loop (31). Although targeting inhibition of SLC7A5/mTORC1 pathway enhances chemosensitivity of tumor cells (30), the role of SLC7A5 in cell cycle progression is still unclear. In this study, overexpression of SLC7A5 significantly increased the phosphorylation status of known DNA damage repair sensors in ATR/ATM pathway and accelerated cell cycle response in response to ETO treatment in RB cells. Thus, we could conclude that SLC7A5 also enhances chemosensitivity *via* promoting DNA damage repair. Recently, activation of mTORC1 also links to DNA damage response (32), and its role in SLC7A5-mediated DNA damage repair needs to be investigated in a future study.

The anticancer activity of most chemotherapeutic drugs relies on the induction of DNA damage in rapidly cycling tumor cells concomitant with the inhibition of cellular DNA repair processes (33). miRNAs targeting proteins involved in these activities also showed therapeutic potential for the treatment of chemoresistant cancer types, and some even proceeded to clinical trials, like miR-16 mimic in progressive mesothelioma and non-small-cell lung cancer (NCT02369198) (34, 35). In this study, we first revealed that enforced miR-184 expression enhances chemosensitivity of RB cells *via* directly targeting SLC7A5. The importance of the current findings stem from the inherent resistance of RB to chemotherapy (3, 4) and the recent attention given to SLC7A5 inhibition, which has been reported with the potential to inhibit tumor growth and metastasis both *in vitro* and *in vivo* (30, 36). JPH203, a novel and selective SLC7A5 inhibitor, is currently being evaluated in a phase 2 clinical trial in patients with advanced biliary tract cancers (UMIN Clinical Trials Registry UMIN000034080) based on the promising results of the phase 1 clinical trial in patients with advanced solid tumors (37). Thus, miR-184 may have the potential to be used as an adjuvant therapeutic agent for RB chemotherapy.

In conclusion, our results have demonstrated for the first time that miR-184 is a tumor-suppressive miRNA in RB and potently enhances chemosensitivity *in vitro*. Through directly targeting SLC7A5, miR-184 inhibits proliferation, migration, and invasion and enhances impairment of DNA damage repair (G2/M phase arrest) and cellular apoptosis of RB cells exposed to chemotherapeutic drugs. Thus, targeting the miR-184/SLC7A5 pathway will provide new opportunities for drug development to reverse chemotherapeutic resistance in RB.

## Data Availability Statement

All datasets generated for this study are included in the article/Supplementary Material.

## Ethics Statement

The studies involving human participants were reviewed and approved by Institutional Review Board of Tongji Hospital (Wuhan, China). Written informed consent to participate in this study was provided by the participants' legal guardian/next of kin.

## Author Contributions

T-GH and J-BC designed the research. J-BC supervised the research and revised the manuscript. Z-YX, H-JY, and HQ conducted the experiments. T-GH, Z-YX, and J-BC collected clinical samples. T-GH and Y-QX analyzed the data. T-GH and Z-YX wrote the manuscript.

### Conflict of Interest

The authors declare that the research was conducted in the absence of any commercial or financial relationships that could be construed as a potential conflict of interest.

## Supplementary Material

Figure S1 (Relates to Figure 2) miR-184 inhibits proliferation, migration and invasion of RB cells. **(A)** miR-184 expression in Y79 and WERI cells transfected with miR-184 mimic, inhibitor, and negative control (NC) was detected by qRT-PCR. **(B)** WERI cells were transfected with miR-184 mimic, inhibitor, and negative control (NC), cell viability was detected by MTT assay. **(C)** Statistical analysis of the EdU-positive cell ratio in WERI cells transfected with miR-184 mimic, inhibitor, and negative control (NC). **(D)** Transwell assay of the migration and invasion ability in WERI cells with different transfection. Scale bar: 100 μm. **(E)** Statistical analysis of the cell numbers through the chamber in WERI cells with different transfection. Data were presented as mean ± SD of three independent experiments. ^*^*P* < 0.05, ^**^*P* < 0.01, ^***^*P* < 0.0001 vs. negative control group.

Figure S2 (Relates to Figure 3) miR-184 increases expression of apoptosis related mRNAs of RB cells in response to ETO treatment. **(A)** Expression of apoptosis related mRNAs in WERI cells transfected with miR-184 mimic, inhibitor or negative control (NC) were detected by qRT-PCR. **(B)** WERI cells were transfected with miR-184 mimic, inhibitor or negative control (NC) together with ETO (0.25 μM) for 48 h, expression of apoptosis related mRNAs was detected by qRT-PCR. Data were presented as mean ± SD of three independent experiments. ^*^*P* < 0.05, ^**^*P* < 0.01, ^***^*P* < 0.0001 vs. negative control group.

Figure S3 (Relates to Figures 5, 6) miR-184 inhibits proliferation, migration, and invasion, while enhances apoptosis and G2/M phase arrest of RB cells in response to ETO treatment via inhibiting SLC7A5. **(A)** Western blot analysis of SLC7A5 expression in Y79 cells and WERI cells transfected with miR-184 mimic alone or together with SLC7A5 expression vector (pcDNA3.1-SLC7A5). **(B)** Statistical analysis of the EdU-positive cell ratio in WERI cells transfected with miR-184 mimic alone or together with SLC7A5 expression vector (pcDNA3.1-SLC7A5). **(C)** Statistical analysis of the cell numbers through the transwell chamber in WERI cells transfected with miR-184 mimic alone or together with SLC7A5 expression vector (pcDNA3.1-SLC7A5). **(D)** WERI cells transfected with miR-184 mimic alone or together with SLC7A5 expression vector (pcDNA3.1-SLC7A5) were treated with ETO (0.25 μM) for 48 h, cellular apoptosis was detected by flowcytometry and the Annexin V^+^PI^+^-positive cell ratio were presented. **(E)** 48 h after transfected with miR-184 mimic alone or together with SLC7A5 expression vector (pcDNA3.1-SLC7A5), Y79 cells were treated with ETO (0.25 μM) for different time and the ratio of Y79 cells in G2/M phase in each time point were presented. Data were presented as mean ± SD of three independent experiments. ^**^*P* < 0.01, ^***^*P* < 0.0001 *vs*. negative control group.
