# Supplementary figures and images for: Tumor Suppressor miR-184 Enhances Chemosensitivity by Directly Inhibiting SLC7A5 in Retinoblastoma
Source: Front Oncol. 2019 Nov 15;9:1163. doi: 10.3389/fonc.2019.01163 (PMC6876683; doi:10.3389/fonc.2019.01163)

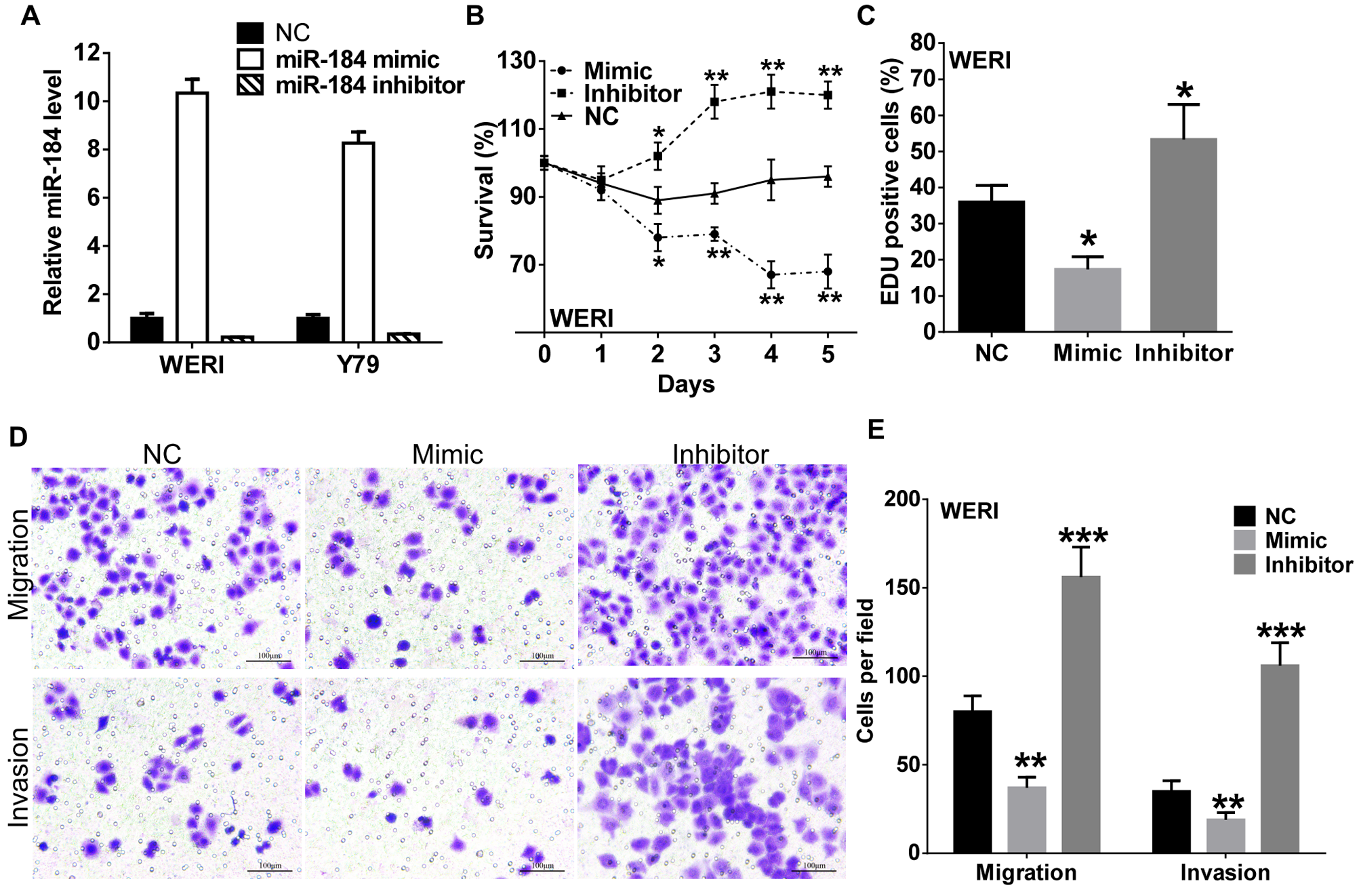

Supplement: Figure S1 — (Relates to Figure 2) miR-184 inhibits proliferation, migration and invasion of RB cells. (A) miR-184 expression in Y79 and WERI cells transfected with miR-184 mimic, inhibitor, and negative control (NC) was detected by qRT-PCR. (B) WERI cells were transfected with miR-184 mimic, inhibitor, and negative control (NC), cell viability was detected by MTT assay. (C) Statistical analysis of the EdU-positive cell ratio in WERI cells transfected with miR-184 mimic, inhibitor, and negative control (NC). (D) Transwell assay of the migration and invasion ability in WERI cells with different transfection. Scale bar: 100 μm. (E) Statistical analysis of the cell numbers through the chamber in WERI cells with different transfection. Data were presented as mean ± SD of three independent experiments. *P < 0.05, **P < 0.01, ***P < 0.0001 vs. negative control group. [file Image_1.TIF]

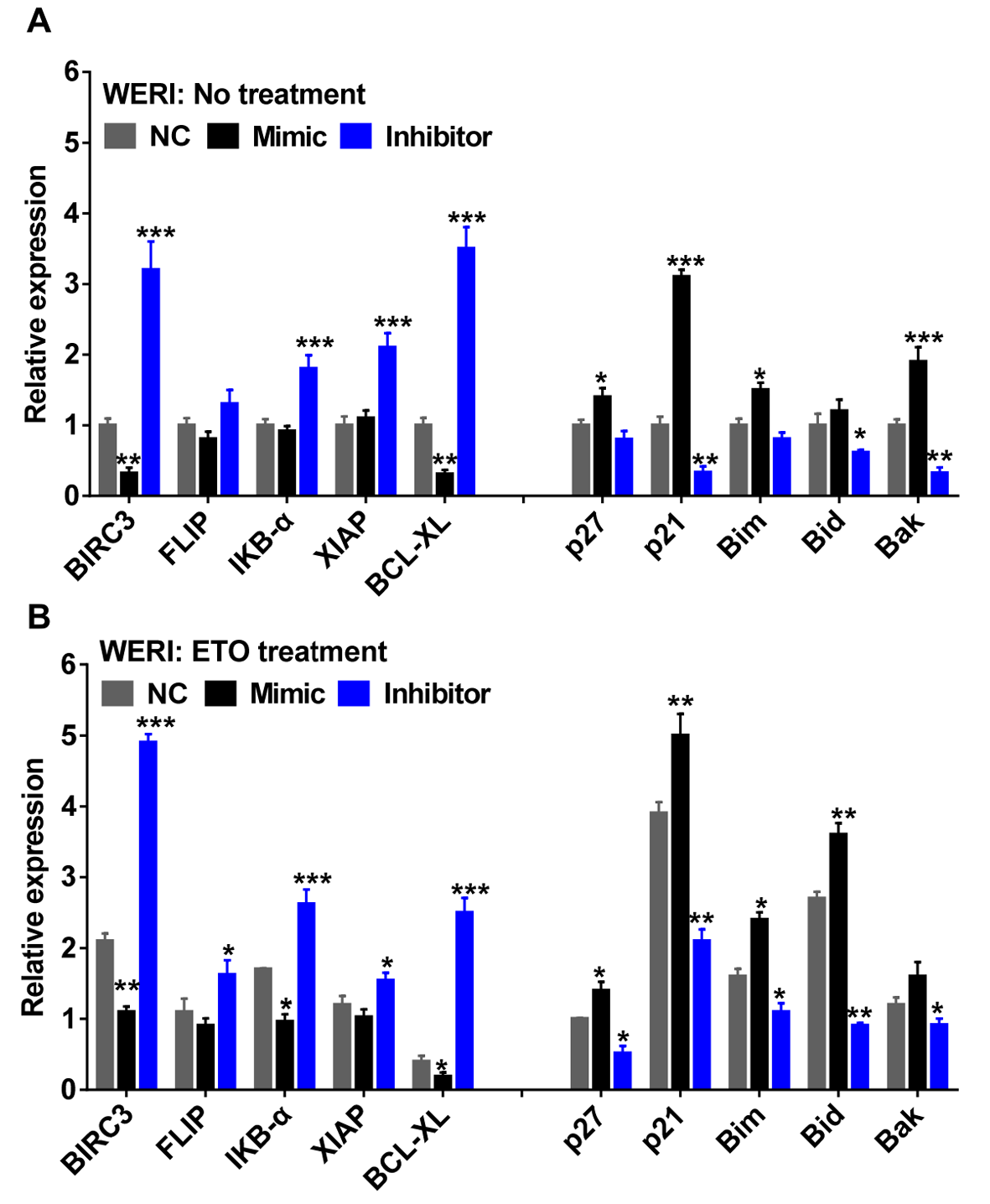

Supplement: Figure S2 — (Relates to Figure 3) miR-184 increases expression of apoptosis related mRNAs of RB cells in response to ETO treatment. (A) Expression of apoptosis related mRNAs in WERI cells transfected with miR-184 mimic, inhibitor or negative control (NC) were detected by qRT-PCR. (B) WERI cells were transfected with miR-184 mimic, inhibitor or negative control (NC) together with ETO (0.25 μM) for 48 h, expression of apoptosis related mRNAs was detected by qRT-PCR. Data were presented as mean ± SD of three independent experiments. *P < 0.05, **P < 0.01, ***P < 0.0001 vs. negative control group. [file Image_2.TIF]

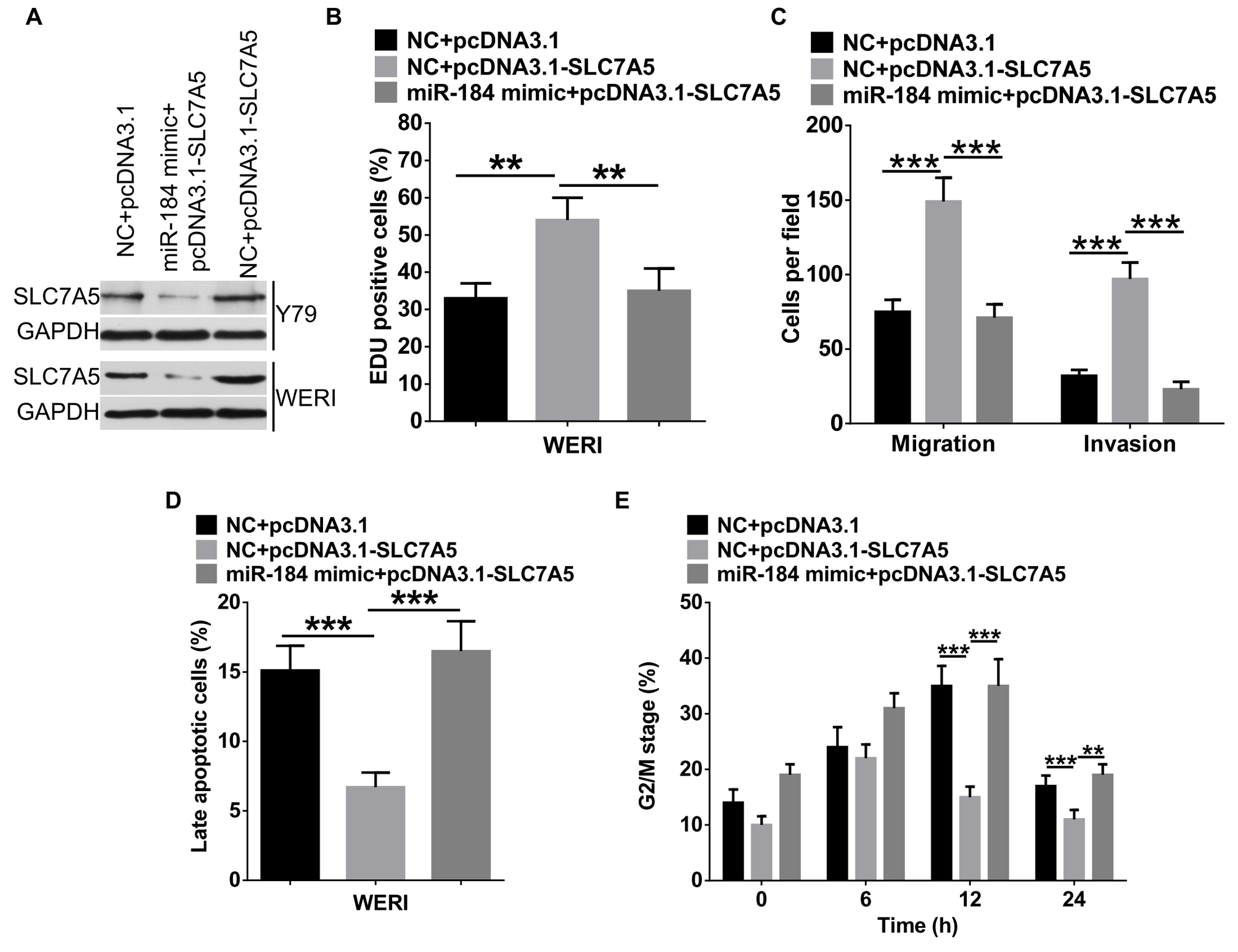

Supplement: Figure S3 — (Relates to Figures 5, 6) miR-184 inhibits proliferation, migration, and invasion, while enhances apoptosis and G2/M phase arrest of RB cells in response to ETO treatment via inhibiting SLC7A5. (A) Western blot analysis of SLC7A5 expression in Y79 cells and WERI cells transfected with miR-184 mimic alone or together with SLC7A5 expression vector (pcDNA3.1-SLC7A5). (B) Statistical analysis of the EdU-positive cell ratio in WERI cells transfected with miR-184 mimic alone or together with SLC7A5 expression vector (pcDNA3.1-SLC7A5). (C) Statistical analysis of the cell numbers through the transwell chamber in WERI cells transfected with miR-184 mimic alone or together with SLC7A5 expression vector (pcDNA3.1-SLC7A5). (D) WERI cells transfected with miR-184 mimic alone or together with SLC7A5 expression vector (pcDNA3.1-SLC7A5) were treated with ETO (0.25 μM) for 48 h, cellular apoptosis was detected by flowcytometry and the Annexin V+PI+-positive cell ratio were presented. (E) 48 h after transfected with miR-184 mimic alone or together with SLC7A5 expression vector (pcDNA3.1-SLC7A5), Y79 cells were treated with ETO (0.25 μM) for different time and the ratio of Y79 cells in G2/M phase in each time point were presented. Data were presented as mean ± SD of three independent experiments. **P < 0.01, ***P < 0.0001 vs. negative control group. [file Image_3.TIF]
